# Minimally invasive surgery for pyeloplasty poised to become The preferred surgical technique irrespective of age

**DOI:** 10.1590/S1677-5538.IBJU.2022.0194.1

**Published:** 2022-11-01

**Authors:** Daniel G. DaJusta

**Affiliations:** 1 Nationwide Children's Hospital Columbus OH USA Nationwide Children's Hospital, Columbus, OH, USA

## COMMENT

For a minimally invasive surgery (MIS) technique to become mainstream and perhaps overtake the open technique as the gold standard it must achieve a couple of goals. First and foremost, it must have a similar or better success rate, after all, adequate treatment of the condition is the main goal. If the success rate is at least similar, it must provide an advantage either by a decrease in the complication rate or the recovery period. Finally, providing a benefit from the cosmetic endpoint can also lead to it becoming the preferable approach. By using a multi-institutional approach, with a large number of patients, the present study highlighted the main reasons why the minimally invasive is becoming the preferable option to perform a pyeloplasty (1). This study showed that the MIS approach both laparoscopically and robotically achieved similar success and complication rates but provided a benefit in the recovery with a shorter hospital length of stay.

While using this multi-institutional approach is a significant strength of the manuscript, it does add one possible weakness. The different institutions likely have different post-operative protocols mostly based on the surgeon's preference. They may or may not be using enhanced recovery after surgery (ERAS) protocols. Furthermore, it is not clear in the manuscript that the techniques were evenly distributed among institutions. These differences could partly influence the findings regarding hospital stay, specifically the fact that robotic technique had a shorter hospital stay than even laparoscopy. It would be expected that both MIS techniques to have similar recovery as shown in a recent single institutional series (2). Nevertheless, the findings in this manuscript described above are on par with another multi-institutional series showing that improved hospital length of stay is associated with the robotic technique (3). These findings appear to also hold even for the younger patients, making MIS for pyeloplasty poised to become the preferred surgical technique irrespective of age.
